# Introducing Upfront Money Can Decrease Discounting in Intertemporal Choices with Losses

**DOI:** 10.3389/fpsyg.2016.01256

**Published:** 2016-08-22

**Authors:** Cheng-Ming Jiang, Hong-Yue Sun, Sheng-Hua Zheng, Liang-Jun Wang, Yu Qin

**Affiliations:** ^1^College of Economics and Management, Zhejiang University of TechnologyHangzhou, China; ^2^Department of Psychology, Shanghai Normal UniversityShanghai, China

**Keywords:** intertemporal choice with losses, intertemporal choice with gains, upfront money effect, discounting rate, salience account

## Abstract

People generally tend to advance gains and postpone losses in intertemporal choice. Jiang et al. (2014) recently showed that adding upfront losses or gains to both smaller and sooner (SS) and larger and later (LL) rewards can decrease people’s discounting. To account for this decrease, they proposed the salience hypothesis, which states that introducing upfront losses or gains makes the money dimension more salient than not, thus increasing people’s preference for LL rewards. Considering that decreasing the discounting of delayed losses is imperative and that most previous studies have focused on intertemporal choices with gains, in the current paper we conducted two experiments and used hypothetical money outcomes to examine whether the effect of upfront money could be extended to intertemporal choices with losses. The results showed that when both SS and LL intertemporal losses were combined with an upfront loss or gain, people’s discounting rate decreased and the preference for the SS option increased. This finding further supports the salience account.

## Introduction

We must frequently make trade-offs in daily decisions between consequences occurring at different points in time, for example, taking a job now vs. receiving a higher education and having a chance at a better job later or buying luxury goods now vs. saving money for retirement. To study such intertemporal choices, researchers typically ask participants to choose between smaller and sooner (SS) and larger and later (LL) rewards (e.g., gaining CNY 3500 in a year vs. gaining CNY 5800 in 3 years). People usually show steep discounting and prefer SS rewards to LL rewards (Frederick et al., 2002; Takahashi, 2005; Estle et al., 2007). Steep discounting of delayed rewards can disrupt the achievement of long-term goals, such as insufficient saving for retirement (Figner et al., 2010; Findley and Caliendo, 2014).

Temporal discounting also occurs in the domain of losses, leading people to postpone the losses until later, although laboratory studies typically find a tendency to discount gains more steeply than losses (Thaler, 1981; Loewenstein, 1988; Frederick et al., 2002; Hardisty and Weber, 2009). Actually, many personal and social issues, such as substance abuse, ecological destruction, excessive borrowing, climate change, among others, are products of discounting delayed gains as well as losses. For example, substance abuse appears frequently in people who relatively focus too much on current pleasures while remaining insensitive to delayed health losses (Myerson et al., 2016). Decreasing the discounting of delayed losses is thus as imperative as that of delayed gains among both individuals and groups. However, most previous studies have focused on how to reduce people’s discounting in intertemporal choices with gains rather than losses. For example, Read et al. (2005) found that framing delays (for example, in 3 months) as dates (for example, on the 1st of October) can make participants more likely to choose LL rewards; Magen et al. (2008) showed that explicitly stating the default outcomes associated with each option (for example, gaining CNY 3500 in a year and CNY 0 in 3 years vs. gaining CNY 0 in a year and CNY 5800 in 3 years) leads participants to choose LL rewards more frequently. Jiang et al. (2014) recently introduced a new way to decrease people’s discounting to delayed rewards, that is, by adding upfront losses as well as gains^1^ to both SS and LL rewards. For example, if an immediate loss or gain of CNY 160 was added to a pairwise choice of “gaining CNY 3500 in a year vs. gaining CNY 5800 in 3 years,” the preference for the LL option (i.e., losing/gaining CNY 160 immediately and gaining CNY 5800 in 3 years) over the SS option (i.e., losing/gaining CNY 160 immediately and gaining CNY 3500 in a year) increased. According to their saliency account, the introduction of upfront money to the SS and LL rewards would enhance the salience of the money dimension and make people assign a greater decision weight to the money dimension. An increased preference for the LL rewards was then detected.

Because there is little important research that examines how to decrease the discounting rate in intertemporal losses compared with intertemporal gains, we investigated in this paper whether the upfront money effect can be extended to the loss domain. Based on the salience account, we assumed that introducing upfront money might make the money dimension more salient and cause people to focus more on the money, therefore increasing people’s preference for the SS options (i.e., the options losing less) in pairwise intertemporal losses. We conducted two experiments to test this assumption. Experiment 1 and Experiment 2 examined whether introducing upfront gains and losses, respectively, to the SS and LL losses could reduce people’s discounting and thus increase the preference for SS options.

## Experiment 1

### Method

In Experiment 1, 181 undergraduates (77 males, two did not report their genders, *M*_age_ = 19.75, *SD* = 0.93) from Zhejiang University of Technology participated in a class for course credit. This study was carried out in accordance with the recommendations of Shanghai Normal University ethical guidelines and the Declaration of Helsinki, with written informed consent from all participants.

Each of the participants was randomly assigned one of two of the following conditions: participants in the pure loss condition responded to simple choice pairs (e.g., losing CNY 210 in a week vs. losing CNY 250 in 5 weeks), whereas those in the upfront money condition had the same choices, except that both the SS and LL options were introduced with the same upfront gains (e.g., gaining CNY 11 now and losing CNY 210 in a week vs. gaining CNY 11 now and losing CNY 250 in 5 weeks). The money outcomes in this experiment were hypothetical (see the Appendix 1).

To assess whether the results were maintained for different losses, each participant in the pure loss condition indicated his or her preferences for two pairwise choices, between which the losses were varied [i.e., Choice 1: losing CNY 210 in a week vs. losing CNY 250 in 5 weeks, Choice 2: losing CNY 3500 in a year vs. losing CNY 5800 in 3 years; these two pairwise choices were both adapted from Experiment 1A in Jiang et al. (2014)] [the loss amounts in Choice 2 are larger than those in Choice 1 while the delays in Choice 2 are longer than those in Choice 1, therefore the two attributes (loss amount and delay length) are correlated^2^]. Correspondingly, the participants in the upfront money condition also completed two pairwise choices (**Table 1**).

**Table 1:** Questionnaire items and summary of the choice results for Experiment 1.

Choice	Item (proportion of responses, 95% confidence interval) (%)		The difference between the proportions of choosing SS options (95% confidence interval) (%)
	Pure loss condition	Upfront money condition	
1	Losing CNY 210 in a week (46.2, 36.3–56.3) vs. losing CNY 250 in 5 weeks (53.8, 43.7–63.7)	Gaining CNY 11 now and losing CNY 210 in a week (61.1, 43.4–72.9) vs. gaining CNY 11 now and losing CNY 250 in 5 weeks (38.9, 50.8–70.5)	15.0 (0.5–28.6)
2	Losing CNY 3500 in a year (48.4, 38.4–58.5) vs. losing CNY 5800 in 3 years (51.6, 41.5–61.6)	Gaining CNY 160 now and losing CNY 3500 in a year (68.9, 58.7–77.5) vs. gaining CNY 160 now and losing CNY 5800 in 3 years (31.1, 22.5–41.3)	20.5 (6.2–33.7)

Each participant indicated his or her preferences for two pairwise choices in a questionnaire, which was presented with other unrelated questions on paper. The order of the two pairwise choices was counterbalanced.

### Results and Discussion

As shown in **Table 1**, for Choice 1, 46.2% of participants chose the SS option in the pure loss condition, whereas in the upfront money condition, 61.6% participants preferred the SS option. For Choice 2, 48.4% of participants chose the SS option in the pure loss condition, whereas 68.9% participants preferred the SS option in the upfront money condition. A random intercept model was fitted using the Stata 11 command “xtlogit,” with choice as dependent variable, and upfront money, loss amount, and upfront money × loss amount as independent variables^3^. The results showed that adding upfront money or not had a significant and negative effect on the preference, *coef.* = -0.93, *z* = -1.97, *p* = 0.048, 95% *CI* (-1.86, -0.01). The loss amount (or the delay length) did not have a significant effect on the preference, *coef.* = -0.13, *z* = -0.37, *p* = 0.71, 95% *CI* (-0.86, 0.59). There was no interaction between the adding money and the loss amount (or the delay length), *coef.* = -0.39, *z* = -0.73, *p* = 0.47, 95% *CI* (-1.44, 0.66). The regression model can be summarized as follows:

Y=0.24−0.93UFM−0.13LA−0.39UFM×LA

(Y represents choice, UFM represents upfront money, LA represents loss amount)

These results suggested that the introduction of upfront gains to both the SS and LL losses significantly reduced people’s discounting and increased the preference for SS losses (from 46.2% to 61.1% in Choice 1; from 48.4% to 68.9% in Choice 2). The effect remained unchanged regardless of whether the losses were small or large (or the delays were short or long). The findings further supported the salience account that adding upfront gains makes the money dimension more salient and leads participants to prefer SS losses more.

## Experiment 2

In Experiment 2, we introduced upfront losses, instead of gains, to both the SS and LL losses to examine whether the effects of upfront losses could also be extended to intertemporal choices involving losses.

### Methods

A total of 195 undergraduates (80 males, one did not report their gender, *M*_age_ = 20.84, *SD* = 2.04) from Zhejiang University of Technology participated in this experiment. The data were collected in the library’s study room. This study was carried out in accordance with the recommendations of Shanghai Normal University ethical guidelines and the Declaration of Helsinki, with written informed consent from all participants.

Each of the participants was randomly assigned to one of two of the following conditions: in the pure loss condition, participants responded to typical choice pairs as in Experiment 1, whereas those in the upfront money condition had the same choices except that both of the SS and LL losses were combined with upfront losses. As in Experiment 1, the money outcomes in this experiment were also hypothetical (see the Appendix 1).

In the upfront money condition, we adopted the logic behind choice stimuli design in Experiments 1B, 2B, and 2C of Jiang et al. (2014). That is, the losses added to SS losses were slightly larger than those added to LL losses (e.g., losing CNY 16 now and losing CNY 210 in a week vs. losing CNY 11 now and losing CNY 250 in 5 weeks), thus making the prospects of SS losses relative to LL losses in the upfront money condition were worse than the prospects of SS losses relative to LL losses in the pure loss condition. Therefore, the SS losses should be less popular in the former condition than those in the latter condition.

To assess whether the results were maintained for different losses, each participant in the pure loss condition indicated his or her preferences for two pairwise choices, between which the losses were varied (i.e., Choice 1: losing CNY 210 in a week vs. losing CNY 250 in 5 weeks, Choice 2: losing CNY 3500 in a year vs. losing CNY 5800 in 3 years; the loss amount and the delay length are correlated in the same way as in Experiment 1). Correspondingly, the participants in the upfront money condition also completed two pairwise choices (**Table 2**).

**Table 2:** Questionnaire items and summary of the choice results for Experiment 2.

Choice	Item (proportion of responses, 95% confidence interval) (%)		The difference between the proportions of choosing SS options (95% confidence interval) (%)
	Pure loss condition	Upfront money condition	
1	Losing CNY 210 in a week (33.3, 24.8–43.1) vs. losing CNY 250 in 5 weeks (66.7, 56.9–75.2)	Losing CNY 16 now and losing CNY 210 in a week (54.2, 44.2–63.8) vs. losing CNY 11 now and losing CNY 250 in 5 weeks (45.8, 36.2–55.8)	20.8 (6.9–33.7)
2	Losing CNY 3500 in a year (50.5, 40.8–60.2) vs. losing CNY 5800 in 3 years (49.5, 39.9–59.2)	Losing CNY 165 now and losing CNY 3500 in a year (65.6, 55.7–74.4) vs. losing CNY 160 now and losing CNY 5800 in 3 years (34.4, 25.6–44.3)	15.1 (1.3–28.2)

Each participant indicated his or her preferences for two pairwise choices in the questionnaire, which was presented with other unrelated questions on paper. The order of the two pairwise choices in each condition was counterbalanced. After completion, each participant received a small gift.

### Results and Discussion

As **Table 2** illustrates, for Choice 1, 33.3% of participants chose the SS option in the pure loss condition, whereas in the upfront money condition, 54.2% participants preferred the SS option. For Choice 2, 50.5% of participants chose the SS option in the pure loss condition, whereas 65.6% participants preferred the SS option in the upfront money condition. As the same as Experiment 1, we used the Stata 11 command “xtlogit” to fit a random intercept model, with choice as dependent variable, and upfront money, loss amount, and upfront money × loss amount as independent variables. The results showed that adding upfront money or not had a significant and negative effect on the preference, *coef.* = -1.15, *z* = -2.83, *p* = 0.005, 95% *CI* (-1.95, -0.35). The loss amount (or the delay length) also had a significant and negative effect on the preference, *coef.* = -0.95, *z* = -2.72, *p* = 0.007, 95% *CI* (-1.64, -0.27). No interaction was detected between the adding money and the loss amount (or the delay length), *coef.* = 0.32, *z* = 0.65, *p* = 0.513, 95% *CI* (-0.63, 1.26). The regression model can be summarized as follows:

Y=0.93−1.15UFM−0.95LA+0.32UFM×LA

(Y represents choice, UFM represents upfront money, LA represents loss amount)

These results suggested that the introduction of upfront losses to both intertemporal options significantly reduced people’s discounting and increased the preference for SS losses (from 33.3% to 54.2% in Choice 1; from 50.5% to 65.6% in Choice 2) even though the prospects of SS losses relative to LL losses in the upfront money condition were worse than the prospects of SS losses relative to LL losses in the pure loss condition. This effect was maintained across different loss sizes. These observations were consistent with the prediction of the salience hypothesis that the effects of upfront losses in intertemporal choices with rewards also can be extended to intertemporal choices with losses. Compared with Experiment 1, we found an extra significant and negative effect of loss amount (or delay length) on the preference, which showed an increased preference for SS losses when the losses in intertemporal choice were small (or delay lengths were shorter). However, because we are not interested in the effects of loss amount (or delay length) on intertemporal choice and we did not strictly manipulate the loss amounts (or the delay lengths), we did not discuss this here.

## General Discussion

Based on the upfront money effect found in intertemporal choices with rewards (Jiang et al., 2014), this paper shows that this effect also extends to intertemporal choices with losses. When upfront losses or gains were added to SS and LL intertemporal losses, people’s discounting rate decreased and the preference for the SS option increased. Laboratory studies of intertemporal choice typically find that the desire to have good things immediately is much stronger than the desire to postpone negative outcomes (Thaler, 1981; Loewenstein, 1988; Frederick et al., 2002; Hardisty and Weber, 2009). Xu et al. (2009) found that the discounting of future losses and the discounting of gains occurs asymmetrically in the brain. Comparing our findings with the results in Jiang et al. (2014), asymmetry in discounting could also be detected. For example, in the choice between losing CNY 210 in a week vs. losing CNY 250 in 5 weeks (choice 1, Experiment 1), only 53.8% of participants chose the delayed loss. Whereas when the choice remained unchanged, except that losses were substituted with rewards, 87.5% of participants chose the early rewards (choice 2, Experiment 1A, Jiang et al., 2014). However, despite the asymmetric gain/loss discounting, the upfront money effect of intertemporal choice with gains can also be extended to intertemporal choice with losses.

Based on the upfront money effect found by Jiang et al. (2014), Sun and Jiang (2015) further hypothesized that, if the salience account was right, the effect of discounting decreasing could be extended to adding dated-money between SS and LL rewards and after LL rewards, because introducing dated money between the SS and LL rewards or after the LL rewards may also make the money dimension more salient and, thus, decrease people’s discounting. Sun and Jiang confirmed their hypothesis. Therefore, to further examine the salience account and to examine whether all types of extra money effects (not only upfront money effect) could be extended to the loss domain, we examined whether the effects in Sun and Jiang could be extended to the intertemporal choice in loss domain. Each of 500 participants was randomly assigned one of five conditions and were offered one set of options: *the pure loss condition*, “Losing CNY 210 in a week (53.0%— the percentage of participants who chose this option) vs. Losing CNY 250 in 5 weeks (47.0%)”; *the intervening gain condition*, “Losing CNY 210 in a week and gaining CNY 11 in 3 weeks (83.0%) vs. Gaining CNY 11 in 3 weeks and Losing CNY 250 in 5 weeks (17.0%)”; *the intervening loss condition*, “Losing CNY 210 in a week and losing CNY 11 in 3 weeks (67.7%) vs. Losing CNY 11 in 3 weeks and Losing CNY 250 in 5 weeks (32.3%)”; *the later gain condition*, “Losing CNY 210 in a week and gaining CNY 11 in 6 weeks (62.6%) vs. Losing CNY 250 in 5 weeks and gaining CNY 11 in 6 weeks (37.4%)”; *the later loss condition*, “Losing CNY 210 in a week and losing CNY 11 in 6 weeks (72.9%) vs. Losing CNY 250 in 5 weeks and losing CNY 11 in 6 weeks (27.1%).” The results showed that the introduction of an intervening loss/gain or a later loss/gain to both intertemporal losses statistically significantly (all *ps* < 0.05, except the later gain condition, *p* = 0.17) increase the participants’ preferences for SS option and reduced people’s discounting, suggesting that the intervening money effect and the later money effect could also be extended to the intertemporal choice in loss domain. The upfront money effect, the intervening money effect and the later money effect in the loss domain in this study, together with these effects in the gain domain in Jiang et al. (2014) and in Sun and Jiang (2015) support the salience account, which states that introducing extra money to pairwise intertemporal options makes the money dimension more salient and thus decreases discounting.

Several possible explanations have been excluded to explain the extra money effects until now, i.e., the normative exponential and descriptive hyperbolic discounting models which agree on the independence and additive assumptions; the integration hypothesis which assumes that decision makers integrate upfront money with final money and make a decision with a bottom line at the end (Jiang et al., 2014), the time scale hypothesis which assumes that the presentation of the 0-delay amount anchors the time dimension at 0 rather than the delivered time of the SS outcomes may make the delay between the LL and SS outcomes appear shorter than when the upfront money is not introduced, and the preference for improvement account that assumes decision makers like outcomes to improve rather than worsen over time (Sun and Jiang, 2015). Although the salience account is consistent with all the current findings about the extra money effects, it does not mean that there are not any other possible accounts that could explain the effects as well. For example, the extra money effects may reflect the sensitivity of relative value of money outcome to contextual changes (Engelmann and Hein, 2013), that is, adding the extra small money made comparison of the value of money outcome between SS and LL options larger than when the extra money is not introduced, thus increased people’s preference for the option with a greater value (LL options in choices between intertemporal gains and SS options in choices between intertemporal losses). Further studies are required to fully examine the salience account and to explore other possible explanations.

Previous studies used hypothetical outcomes to explore the extra money effects (Jiang et al., 2014; Sun and Jiang, 2015). In the current study, we also asked the participants to choose between hypothetical money outcomes, rather than delivering the actual money outcomes, because executing real losses (e.g., CNY 3500) with the participants was impossible. Although it was found that using hypothetical intertemporal money outcomes yield the same results as using actual money outcomes (Johnson and Bickel, 2002; Madden et al., 2004; Bickel et al., 2009), future studies were required to test the extra money effects with actual money outcomes.

Every day we make decisions that involve trade-offs between short-term and long-term consequences that include gains and positive events as well as losses and negative events across single-dated outcomes as well as multiple-dated outcomes. However, most previous studies focus on intertemporal choices with gains and with single-dated outcomes (e.g., Green and Myerson, 2004; Kable and Glimcher, 2007; Wittmann et al., 2007). This paper may shed some light on the underlying mechanism of intertemporal choices with losses and multiple-dated outcomes and provide some insight into a way to reduce people’s discounting of delayed losses.

## Author Contributions

Conceived and designed the experiments: C-MJ and H-YS. Performed the experiments: S-HZ, L-JW, and YQ. Analysed the data: S-HZ, L-JW, and YQ. Interpreted the data: C-MJ, H-YS, and S-HZ. Drafted the paper: C-MJ and H-YS. Revised the paper: C-MJ, H-YS, S-HZ, L-JW, and YQ.

## Conflict of Interest Statement

The authors declare that the research was conducted in the absence of any commercial or financial relationships that could be construed as a potential conflict of interest.

## Footnotes

1 The upfront gain effect was first reported by Urminsky and Kivetz (2011).

2 Thanks to the reviewer for pointing it out.

3 Thanks to the reviewer for suggesting using this method.
