# Supplementary material for: Introducing Upfront Money Can Decrease Discounting in Intertemporal Choices with Losses
Source: Front Psychol. 2016 Aug 22;7:1256. doi: 10.3389/fpsyg.2016.01256 (PMC4992676; doi:10.3389/fpsyg.2016.01256)
Supplement: Supplementary file 1 [file Data_Sheet_1.DOCX]

**Appendix 1**

**The intertemporal choice scenarios of Experiment 1:**

Welcome to participate in the survey of behavioral decision making. The purpose of this survey is to explore people’s propensity in behavioral decision making. There are no right or wrong answers, please be assured that all information will be kept confidentially.

I. Suppose that you face the following pairwise options, which one will you choose, please write "√" to indicate your choice.

A. Losing CNY 210 in a week (pure loss condition)/ Gaining CNY 11 now and losing CNY 210 in a week (upfront money condition)

B. Losing CNY 250 in five weeks (pure loss condition)/ Gaining CNY 11 now and losing CNY 250 in five weeks (upfront money condition)

II. Suppose that you face the following pairwise options, which one will you choose, please use "√" to indicate your choice.

A. Losing CNY 3500 in a year/Gaining CNY 160 now and losing CNY 3500 in a year (upfront money condition)

B. Losing CNY 5800 in three years/Gaining CNY 160 now and losing CNY 5800 in three years (upfront money condition)

**The intertemporal choice scenarios of Experiment 2:**

Welcome to participate in the survey of behavioral decision making. The purpose of this survey is to explore people’s propensity in behavioral decision making. There are no right or wrong answers, please be assured that all information will be kept confidentially.

1. Suppose that you face the following pairwise options, which one will you choose, please write "√" to indicate your choice.

A. Losing CNY 210 in a week (pure loss condition)/ Losing CNY 16 now and losing CNY 210 in a week (upfront money condition)

B. Losing CNY 250 in five weeks (pure loss condition)/ Losing CNY 11 now and losing CNY 250 in five weeks (upfront money condition)

2. Suppose that you face the following pairwise options, which one will you choose, please use "√" to indicate your choice.

A. Losing CNY 3500 in a year/ Losing CNY 165 now and losing CNY 3500 in a year (upfront money condition)

B. Losing CNY 5800 in three years/ Losing CNY 160 now and losing CNY 5800 in three years (upfront money condition)
